# Case Report: Optical Coherence Tomography Usage for Treatment of the Chronically Lost Stent in the Left Main Coronary Artery

**DOI:** 10.3389/fcvm.2022.825542

**Published:** 2022-02-10

**Authors:** Tomislav Krcmar, Ivana Grgic Romic, Vjekoslav Tomulic, Tomislav Jakljevic, Luka Bastiancic, Ivan Zeljkovic

**Affiliations:** ^1^Deparment of Cardiology, Rijeka University Hospital Centre, Rijeka, Croatia; ^2^Department of Cardiology, Sestre Milosrdnice University Hospital Centre, Zagreb, Croatia

**Keywords:** optical coherence tomography, intravascular imaging, stent loss, left main, crush technique, percutaneous coronary intervention (complex PCI)

## Abstract

Acute adverse outcomes of a stent loss during percutaneous coronary intervention (PCI) are well described, however, data on long-term consequences are scarce, especially with intravascular imaging. We report a case of a coronary stent loss in the left main and ostial left circumflex artery (LCx) bifurcation and its migration into the LCx ostium during PCI procedures. This rare complication, which was not immediately noticed, was verified and successfully resolved 5 months after using optical coherence tomography and right trans-radial access. Considering the infrequency of this complication, few cases have been reported, however, our case has several distinct specificities. We aim to encourage the crushing technique in cases of chronic stent loss when the retrieval is not an option and highlight the optical coherence tomography (OCT) value in imaging and evaluation of similar complex settings.

## Introduction

Unnoticed loss of an unexpanded stent during percutaneous coronary intervention (PCI) is a rare and potentially fatal complication, with decreasing incidence in recent years (1–3). A number of mechanisms for stent loss have been suggested, mostly involving coronary anatomy and technical procedural specificities (3–5). Acute adverse outcomes are well described, however, data on long-term consequences of chronically lost stent is scarce, especially with intravascular imaging (1, 5, 6).

We report a case of an unnoticed coronary stent loss in the left main and ostial left circumflex artery (LCx) bifurcation during a primary PCI, which was successfully treated after 5 months with the usage of optical coherence tomography (OCT).

## Case Description

**Figure 1:**
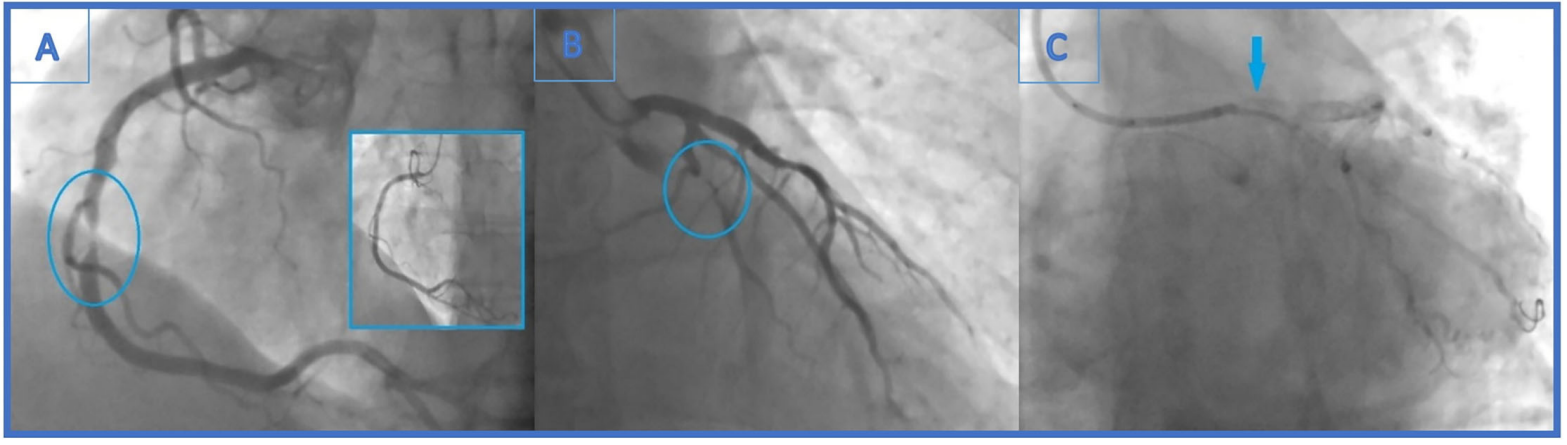
**(A)** Urgent coronary angiography with significant mid-right coronary artery stenosis (99%)-the culprit lesion and final angiographic result in the little window. **(B)** Distal left circumflex artery (LCx) lesion left unsolved (90% stenosis). **(C)** Angiogram showing stent falling from the balloon and darting into the LCx ostium. It is seen as linear radio-opaque object in the LM/LCx.

A 48-year-old male patient, a smoker without a history of chronic diseases, presented with an acute myocardial infarction without ST elevation (NSTEMI) to a local hospital. Urgent coronary angiography demonstrated sub-occlusion (99% stenosis) in the midportion of the dominant right coronary artery (RCA) (Figure 1A) and significant distal LCx (90% stenosis) lesion (Figure 1B).

Primary percutaneous coronary intervention (PCI) was undertaken using a Judkins-right 4 cm guiding catheter with side holes (Cordis, Milpitas, CA, USA). Employing a 0.014 in balanced middleweight (BMW) guidewire (Abbott Vascular, Santa Clara, CA, USA), drug eluting stent (DES) Xience Prime (3.5 × 15 mm, Abbott Vascular) was successfully implanted in a culprit RCA lesion (Figure 1A). Intervention was pursued on the distal LCx lesion with 6 French (Fr) Extra backup guiding (EBU) catheter 3.75 cm (Medtronic, Minneapolis, MN, USA) and BMW guidewire (Abbott Vascular). After predilatation with non-compliant (NC) balloons (Traveler 1.20 × 8 mm and 2.0 × 8 mm, Abbot Vascular), Xience Prime stent (3.0 × 12 mm, Abbot Vascular) could not cross the lesion and the intervention was aborted. Echocardiographic examination showed mildly hypertrophic myocardium with no regional wall abnormalities and left ventricular ejection fraction of 60%. After 3 days of uneventful hospitalization, the patient was released.

**Figure 2:**
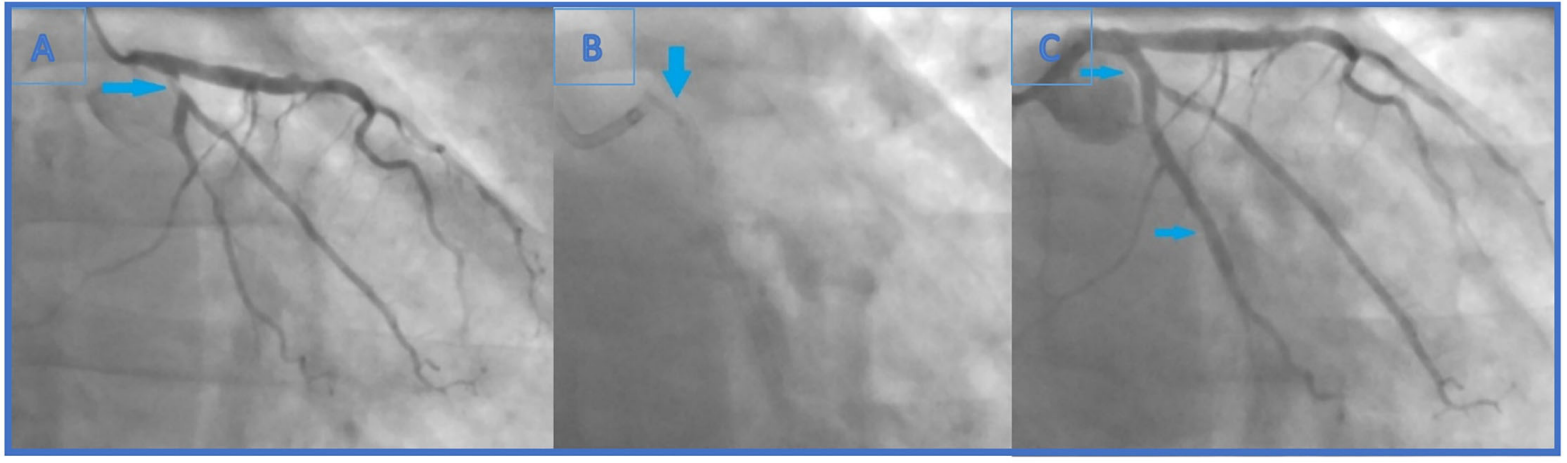
**(A)** Second coronary angiography revealed newly formed, highly significant ostial (95%) (Medina 0.0.1) LCx stenosis. Also, previously known distal LCx stenosis (90%) was shown. **(B)** The end of the second intervention (angiography without iodinated contrast): the lost stent in the left main coronary artery (arrow). The distal part of the lost stent (part in the LCx, 70% of its length) is crushed with the proximal LCx stent. **(C)** The end of the second intervention where proximal and distal LCx stenosis were solved (arrows indicating lesion sites where the stents were implanted).

After 4 months, the patient reported effort angina (Canadian Cardiac Society—CCS class 2), and the stress-ECG test showed signs of ischaemia. Coronary angiography revealed newly formed, highly significant ostial (95%) LCx stenosis (LM 0.0.1 lesion according to Medina classification) (Figure 2A). Previously known distal LCx stenosis (90%) was also shown.

Intervention was pursued through 6F EBU catheter 3.5 cm (Medtronic). The first obtuse marginal artery (OM1) and LCx were wired with BMW (Abbot Vascular) in OM1 and BMW Universal II (Abbot Vascular) in LCx. Distal LCx lesion was pre-dilatated with non-compliant (NC) balloons (Traveler 2.5 × 15 mm and 2.0 × 20 mm, Abbot Vascular), after which Resolute Onyx stent (2.75 × 26 mm, Medtronic) was placed. Ostial LCx lesion was predilated [up to 22 atmosphere (atm)] with an NC balloon (Traveler 2.5 × 15 mm, Abbot Vascular) and Resolute Onyx stent (3.0 × 12 mm, Medtronic) was implanted in proximal LCx (Figure 2C).

Carefully observing the final result, a linear radiopaque object was seen in the distal segment of the left main coronary artery (LMCA), which has not been verified before. At that point, the operator suspected that the ostial LCx stent had crushed an earlier lost stent which was protruding from the proximal LCx to the distal LMCA (Figure 2B). Afterwards, the first angiography film was viewed, and it was seen how the stent went loose from the wire and darted into the LCx ostium (Figure 1C). Further evaluation was indicated, but this time in our facility using an intravascular imaging modality with the idea to precisely visualize lost stent, its position, and endothelialisation.

The patient came back to our facility 1 month after the second coronary angiography due to lack of the OCT catheter (caused primarily by financial reasons), and without an urgent indication since there was no angiographic or clinical sign of coronary blood-flow obstruction. The right radial artery was used for introducing the OCT catheter (Dragonfly DUO Imaging Catheter, St. Jude Medical, Sylmar, CA, USA) into the coronary arteries. Coronary stents in the proximal LCx and in the mid-RCA were found completely patent. The lack of OCT imaging prior to the first LCx PCI is a limitation as it precluded optimal visualization of the complication.

**Figure 3:**
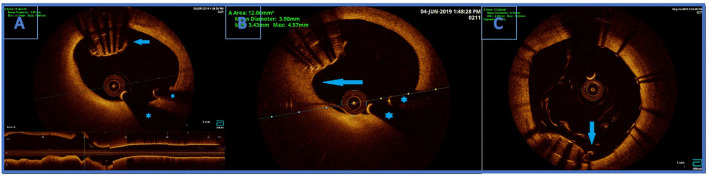
**(A)** Optical coherence tomography (OCT) finding an undeployed part of the lost stent in the left main artery. There are no signs of thrombosis around the stent. Below is the longitudinal view of the left main OCT. ^*^Wire artifacts. **(B)** Optical coherence tomography image. Partially endothelialised (arrow) part of the undeployed stent in the LM. **(C)** OCT showing good apposition of the new stent and a successfully crushed part of the undeployed stent. Arrow indicating a single non fully apposed strut.

OCT visualised undeployed stent protruding (approximately 50% of the stent) from the ostium of LCx to distal segment of LMCA, not causing a significant stenosis (Figure 3A). The lost stent was partially endothelialised (proximal 50% in the LMCA, distal part in the LCx was partially crushed 1 month earlier) (Figure 3B). There was no sign of thrombus formation around or inside the lost stent. Since the distal part of the lost stent was crushed with a stent deployed into a proximal LCx segment during the second coronary intervention, stent deployment from the LMCA to the left anterior descending coronary artery (LAD) with crushing of both the proximal 50% of lost stent and a small part of protruding proximal LCx stent was planned. Using a 7 Fr EBU catheter 3.5 cm Launcher (Medtronic), the stenosis was crossed with a 0.014 in guidewire (Hi-Torque BMW Universal II, Abbot Vascular) and positioned to the distal segment of LAD. “Ultra-thin strut” DES Orsiro (4.0 × 22 mm, Biotronik, Lake Oswego, OR, USA) was deployed into the LMCA ostium to proximal LAD with a mini crush of proximal LCx stent and protruding LCx undeployed stent struts. Then, a Runthrough Hypercoat guidewire (Terumo Europe NV, Lueven, Belgium) was crossed through stent struts in the proximal LCx. After struts dilatation (up to 22 atm) with a semi-compliant (SC) balloon (Maverick 2.5 × 15 mm, Boston Scientific, Marlborough, MA, USA), an additional kissing balloon post dilatation was necessary. It was performed with a semi-compliant (SC) balloon (14 atm) (Maverick 3.0 × 15 mm, Boston Scientific) in LAD and an NC balloon (12 atm) (Emerge 2.75 × 8 mm, Boston Scientific Corp., Natick, MA, USA) in LCx, resulting in the lost stent being crushed between the LAD wall and the new stent in LMCA/LAD.

**Figure 4:**
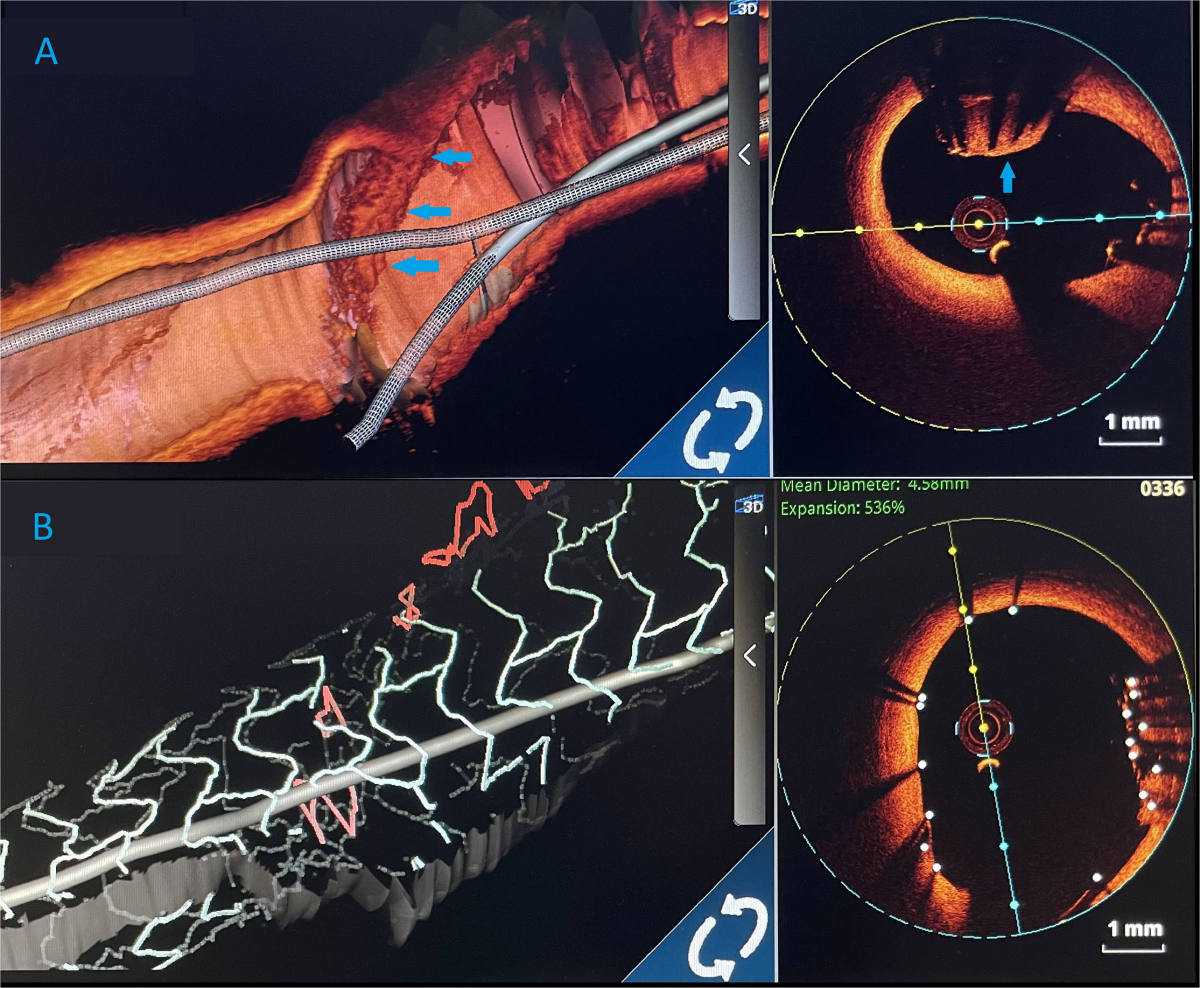
Three-dimensional (3D) images. **(A)** 3D render of an optical coherence tomography (OCT) showing an undeployed lost stent (blue arrows) in the left main artery. **(B)** Optical coherence tomography 3D view. After LM stent implantation, lost stent (blue arrows) is successfully crushed against the left main artery wall.

The procedure was finalized with a proximal optimisation technique (POT) in LMCA with an NC balloon (Quantum 4.5 × 8 mm, Boston Scientific). Final angiography showed TIMI III flow (Figure 4) and OCT demonstrated well-apposed stent struts with a crushed lost stent in LMCA (Figure 3C; Supplementary Video with final OCT pullback from LAD to LM). The patient had an uneventful recovery and is still in follow-up. Dual antiplatelet therapy with ticagrelor (90 mg two times daily) and acetylsalicylic acid (100 mg one time daily) was continued for 12 months after the intervention. Currently, the patient is prescribed ticagrelor (60 mg two times daily) together with acetylsalicylic acid (100 mg once daily) for an additional 12 months. Afterwards, the plan is to recommend acetylsalicylic acid lifelong (100 mg once daily) and a vascular dose of rivaroxaban (2.5 mg two times daily).

## Discussion

We presented a case of a coronary stent loss and its migration into the LCx ostium during an attempt of its distal segment revascularisation. A rare complication was not immediately noticed by the operator, but was OCT verified 5 months later and was successfully resolved using the right trans-radial access. Considering the infrequency of this complication, few cases have been reported in the literature, however, our case has several distinct specificities (1–7).

Firstly, the lost stent was located in the LMCA, a rare location described in only several cases so far (1, 5, 7). More importantly, this was a chronic stent loss, while earlier cases were describing acute management of stent loss in LMCA (1–4, 7, 8). In addition, most reports describe management of acutely noticed and treated stent losses, while in our case, long-term consequences are seen, which are not well known (2, 3, 5, 7).

Secondly, it is generally advised to avoid stent crush or deployment within the LMCA or at a bifurcation lesion for the sake of the restenosis risk (1, 9). However, retrieval of the lost stent was not an option in our case since the distal half was already crushed with the stent deployed into the proximal LCx segment 1 month before. Retrieval of the lost stent can be efficacious with various non-surgical methods, including the small-balloon technique, which is the most frequently used one, double-wire technique, and loop-snare technique (1, 2, 4, 9, 10). Hence, new stent deployment from LMCA to LAD, with crushing of both proximal 50% of the lost stent and a small part of stent protruding to proximal LCx, was planned. The operator considered that the option of stenting from LAD to the LM is a better option than the stenting from LCx. The reason is that in case of stenting from LCx to LM, part of proximal LCx would have four layers of stents (lost stent crushed earlier, ostial LCx stent implanted during second procedure, and a new stent from LCx to the LM). Also, the LCx artery is narrower than the LM and LAD, hence the result of post dilatation and POT would be less optimal.

Brilakis et al. suggested that in cases in which the lost stent cannot be retrieved, deploying or crushing the stent may be a good alternative with good clinical outcomes (4). In another study, lost stents were crushed against the vessel wall in two patients with no major cardiac complications (9).

Thirdly, according to a retrospective study, patients who encountered stent loss had a higher incidence of bleeding, requiring transfusion and causing a need for coronary artery bypass surgery, both not noted in our case (2).

Fourthly, in most cases, the stent was lost due to not crossing the stenosis and being damaged while trying, hence becoming dissociated from its delivery system (1, 4, 10, 11). In our case, one can only speculate that the stent was lost because of the high friction caused by vessel angulation and inadequate guiding of the catheter position. In addition, the operator was not aware of this happening during the initial procedure. Thus, operators should be reminded to check the complete condition of the stent system after unsuccessful deployment, especially if this was the case due to the (heavy) calcification of the lesion and/or anatomical specificities (tortuosity, angulation >90°, bi/trifurcation, etc.) (1–4, 10, 11). Interestingly, 5 months after its loss, the stent was only partially covered by intima with no signs of thrombus formation. This could be explained by dual antiplatelet therapy usage and a normal left ventricle ejection fraction (LVEF 60%) (5–9, 12). The reason the stent could not cross during the initial intervention but did cross at the time of the recurrent event is probably due to: better lesion preparation, more experienced operator, and the partial endothelialisation of the lost stent. We chose OCT over intravascular ultrasound (IVUS) because of its superior resolution imaging, giving a better characterization of neointimal tissue (6, 13, 14). Finally, besides the risk of stent crushing in the critical coronary segment, this complex coronary intervention was entirely performed using only right radial access, while most other similar cases were using femoral access (1–12).

In conclusion, we encourage the crushing technique in cases of chronic stent loss when the retrieval is not an option, along with highlighting the OCT value in imaging and evaluation of similar complex settings. This complex procedure can be done using a transradial approach without affecting the safety or efficacy. Operators should be reminded to check the complete condition of the stent system after an unsuccessful deployment, regardless of the reason.

## Data Availability Statement

The original contributions presented in the study are included in the article/Supplementary Material, further inquiries can be directed to the corresponding author/s.

## Ethics Statement

Ethical review and approval was not required for the study on human participants in accordance with the local legislation and institutional requirements. The patients/participants provided their written informed consent to participate in this study. Written informed consent was obtained from the individual(s) for the publication of any potentially identifiable images or data included in this article.

## Author Contributions

TK performed the intervention together with VT and LB. IG and TJ drafted the manuscript. IZ contributed to manuscript design, drafting, and critical revision. All authors have critically read and reviewed this article, approved the version to be published, and agreed to be accountable for all aspects of the work in ensuring that questions related to the accuracy or integrity of any part of the work are appropriately investigated and resolved.

## Conflict of Interest

The authors declare that the research was conducted in the absence of any commercial or financial relationships that could be construed as a potential conflict of interest.

## Publisher's Note

All claims expressed in this article are solely those of the authors and do not necessarily represent those of their affiliated organizations, or those of the publisher, the editors and the reviewers. Any product that may be evaluated in this article, or claim that may be made by its manufacturer, is not guaranteed or endorsed by the publisher.
